# Supplementary material for: Comprehensive assessment of multiple biases in small RNA sequencing reveals significant differences in the performance of widely used methods
Source: BMC Genomics. 2019 Jun 21;20:513. doi: 10.1186/s12864-019-5870-3 (PMC6588940; doi:10.1186/s12864-019-5870-3)

Influence of estimated secondary structure on accuracy error

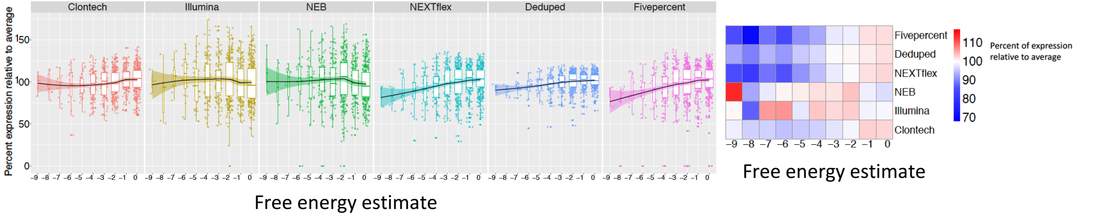

Influence of the identity of the last 2 bases in synthetic sequences on accuracy error

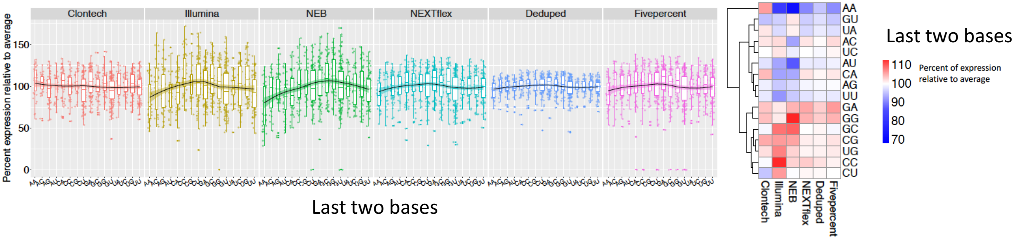

Influence of the identity of the first 2 bases in synthetic sequences on accuracy error

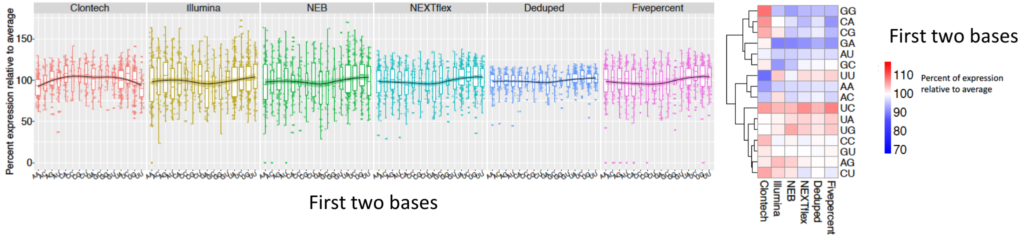

Influence of the number of Cs in synthetic sequences on accuracy error

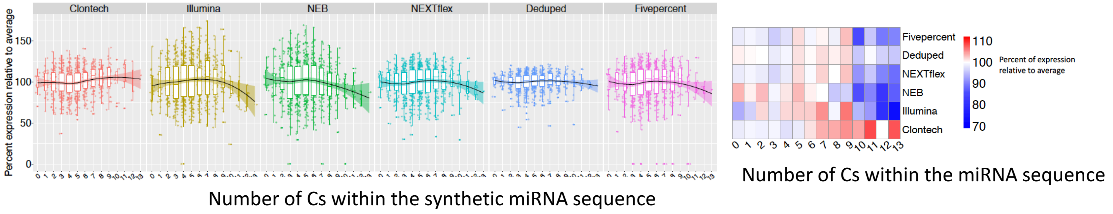

Supplement: Supplementary file 3 — Figure S1. Boxplots and heatmaps of the influence of various synthetic sequence aspects on accuracy error. Boxplots show sequences grouped by various sequence aspects, including: Gibb’s free energy secondary structure estimates, the identity of the last 2 bases, the identity of the first 2 bases, and the number of Cs in the sequence. The percent expression relative to the mean of all synthetic sequences is plotted for each of the 962 synthetic sequences. Heatmaps also depict the percent expression of synthetic sequences grouped by the sequence aspect of interest relative to the mean expression of all the synthetic sequences. Overall, most methods showed a positive quantification relationship with higher or less negative Gibb’s free energy estimates. Most methods showed consistent quantification despite the identity of the last two bases, however NEB and Illumina showed more inconsistent quantification. All of the methods showed fairly consistent quantification despite the identity of the first two bases, however the Deduped method showed more consistent quantification. Most of the methods showed decreased quantification with increasing numbers of Cs, however the Clontech method showed the opposite relationship, and the Deduped method was quite consistent. (PDF 663 kb) [file 12864_2019_5870_MOESM3_ESM.pdf]
