# Supplementary material for: Comprehensive assessment of multiple biases in small RNA sequencing reveals significant differences in the performance of widely used methods
Source: BMC Genomics. 2019 Jun 21;20:513. doi: 10.1186/s12864-019-5870-3 (PMC6588940; doi:10.1186/s12864-019-5870-3)

Examples of miRNAs detected less than average

mmu-miR-540-5p/rno-miR-540-5p
..(((((((.....)))))))..
Minimum free energy
= -7.50 kcal/mol

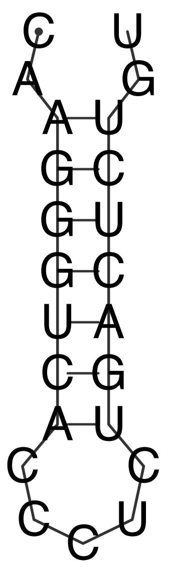

hsa-miR-614
...((((((.......))))))).
Minimum free energy
= -8 kcal/mol

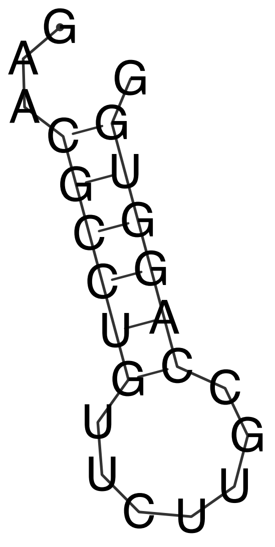

Examples of miRNAs detected more than average

mmu-miR-654-5p
.........(((.....)))..
Minimum free energy
= -0.40 kcal/mol

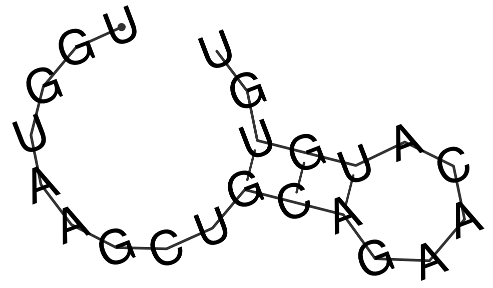

rno-miR-743b
....
Minimum free energy
= 0 kcal/mol

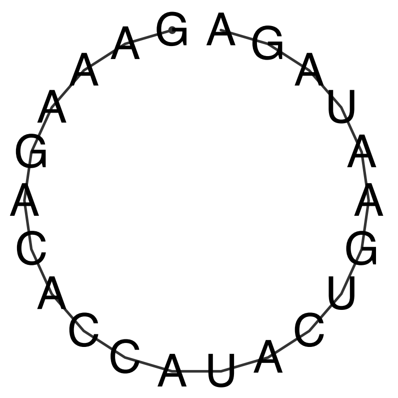

Supplement: Supplementary file 4 — Figure S2. This figure depicts the secondary structure of example of synthetic miRNAs with differences in detection that may be due to secondary structure. Mmu-miR-540-5p/rno-miR-540-5p and hsa-miR-614 were detected less than the average synthetic sequence by all tested methods, while mmu-miR-654-5p and rno-miR-743b were detected more than the average synthetic sequence by all tested methods. (PDF 36 kb) [file 12864_2019_5870_MOESM4_ESM.pdf]
