# Supplementary material for: Comprehensive assessment of multiple biases in small RNA sequencing reveals significant differences in the performance of widely used methods
Source: BMC Genomics. 2019 Jun 21;20:513. doi: 10.1186/s12864-019-5870-3 (PMC6588940; doi:10.1186/s12864-019-5870-3)
Supplement: Supplementary file 9 — Figure S3. Detection of miRNAs and isomiRs and detection consistency of miRNA and isomiRs across various starting amounts. For the detection plots, the number of miRNAs detected above 10 normalized counts and the number of isomiRs detected above 100 normalized counts in all triplicates of batch 1 for each method is plotted on the y-axis. The starting total RNA amount is indicated on the x-axis in nanograms. Only starting amounts in the range of suggested inputs were tested for each method. For the consistency of detection plots, the percentage of miRNAs or isomiRs detected above the threshold by a single triplicate that were not detected above the threshold by the other two triplicates is plotted on the y-axis. The starting total RNA amount is indicated on the x-axis in nanograms. The relationship between the percentage of inconsistently detected miRNAs or isomiRs and starting amount is plotted as a line using a locally estimated scatterplot smoothing regression (LOESS). (PDF 2586 kb) [file 12864_2019_5870_MOESM9_ESM.pdf]

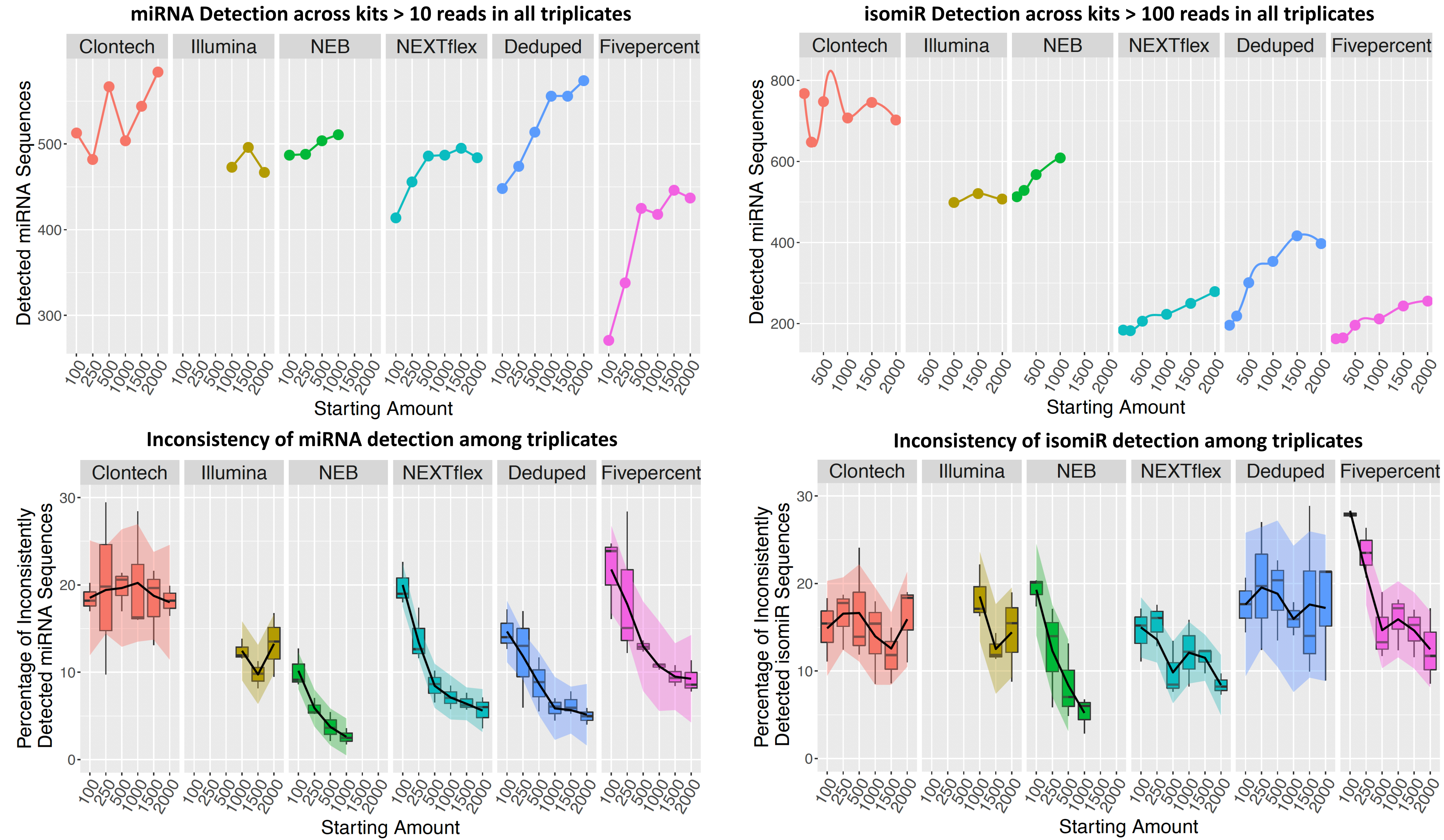

miRNA Detection across kits > 10 reads in all triplicates
isomiR Detection across kits > 100 reads in all triplicates
Inconsistency of miRNA detection among triplicates
Inconsistency of isomiR detection among triplicates
Clontech
Illumina
NEB
NEXTflex
Deduped
Fivepercent
Detected miRNA Sequences
Percentage of Inconsistently Detected miRNA Sequences
Percentage of Inconsistently Detected isomiR Sequences
Starting Amount
100
250
500
1000
1500
2000
300
400
500
200
600
800
0
10
20
30
